# Evaluation of enterotoxin gene expression and enterotoxin production capacity of the probiotic strain *Bacillus toyonensis* BCT-7112^T^

**DOI:** 10.1371/journal.pone.0214536

**Published:** 2019-04-25

**Authors:** Amir Abdulmawjood, Jens Herrmann, Susanne Riede, Guillermo Jimenez, Andre Becker, Gerhard Breves

**Affiliations:** 1 Institute of Food Quality and Food Safety, Research Center for Emerging Infections and Zoonoses (RIZ), University of Veterinary Medicine Hannover, Foundation, Hannover, Germany; 2 Department of Physiology, University of Veterinary Medicine Hannover, Foundation, Hannover, Germany; 3 Rubinum, S.A., Rubi, Spain; Bharathidasan University, INDIA

## Abstract

The aim of the present study was to evaluate the safety of the probiotic strain *Bacillus toyonensis* BCT-7112^T^ (active ingredient of Toyocerin) in relation to the enterotoxins haemolysin BL (Hbl) and the non-haemolytic enterotoxin (Nhe) by performing a quantitative reverse transcription (RT) real-time polymerase chain reaction (PCR) and a Western blot assay. The expression levels of the enterotoxin genes *hblA*, *hblD*, *nheA*, *nheB* and *nheC*, determined by means of RT real-time PCR in *B*. *toyonensis*, were lower than those in *B*. *cereus* reference strains. No expression of *hblC* was detected. The Western blot assays of native and 25-fold concentrated supernatants from *B*. *toyonensis*, using monoclonal antibodies directed against the Hbl component L1 and the Nhe component NheB, showed weak bands. The NheC component was not detected in the native supernatant, but weakly in the 25-fold concentrated supernatant. According to the results of the present study, the enterotoxin expression and protein levels of *B*. *toyonensis* BCT-7112^T^ were absent or clearly lower compared to the *B*. *cereus* reference strains. Thus, their ability to form functional enterotoxins can also be considered to be lower or unlikely compared to the *B*. *cereus* reference strains. This experimental approach can be implemented when studying the health and safety as well as harmlessness of probiotic microorganisms.

## Introduction

*Bacillus cereus* is well known as a spore-forming, food-associated bacterium which is able to produce enteropathogenic toxins. Besides the cytotoxin K [1], two major enterotoxin complexes are known to be causative for food poisoning induced by *B*. *cereus*, namely the haemolysin BL (Hbl) and the non-haemolytic enterotoxin (Nhe) complexes which causes diarrohea. Hbl consists of the protein components B, L1 and L2 [2, 3], encoded by *hblA* (B), *hblC* (L_2_) and *hblD* (L_1_) [2]. Nhe is composed of the protein constituents NheA, NheB and NheC encoded by *nheA*, *nheB* and *nheC* [4]. For both enterotoxin complexes, the combination of their respective components is necessary in order to develop their enterotoxigenic potential [5, 6].

The probiotic strain *B*. *toyonensis* BCT-7112^T^ (NCIMB 14858^T^) is the active ingredient of the feed additive preparation Toyocerin (product of Rubinum S.A.), designated as the type strain [7, 8]. The genome of *B*. *toyonensis* BCT-7112^T^ (NCBI Acc.-No.: CP006863.1) shows relevant differences from the genome of *B*. *cereus* food-poisoning strains regarding the presence of genes considered to be the causative agents of gastrointestinal diseases, e.g., haemolysin BL (Hbl), non-haemolytic enterotoxin (Nhe), cytotoxin K (CytK) and cereulide [9]. The Nhe (non-haemolytic enterotoxin) and Hbl (haemolysin BL) operons present in the genome of *B*. *toyonensis* may be nonfunctional, since some of their toxin components show differences in the corresponding amino acid sequences when compared to those present in *B*. *cereus* food-poisoning strains. Furthermore, only three of the seven genes comprising the operon encoding cereulide synthetase are present in the genome of *B*. *toyonensis*. Therefore, the cereulide synthetase is not expected to be functional. Additionally, the *cytK* gene is absent in the genome of *B*. *toyonensis* [7]. Although the presence of the Nhe and Hbl operons is not synonymous with their expression or functional properties of the gene products *per se* [9, 10], the occurrence of enterotoxin components should be proven at protein level to further assess the toxigenic potential of *B*. *toyonensis* BCT-7112^T^. Thus, the present study aimed to establish and to perform a real-time PCR and a Western blot assay in order to evaluate the ability of *B*. *toyonensis* BCT-7112^T^ (NCIMB 14858^T^) to express and to secrete the components of the enterotoxins Hbl and Nhe on the mRNA and at protein level.

## Material and methods

### Bacterial strains and growth conditions in the DNA and protein analyses

In addition to the probiotic strain *B*. *toyonensis* BCT-7112^T^ (NCIMB-14858^T^) (obtained from RUBINUM S.A., Rubi, Barcelona, Spain), *Bacillus cereus* DSM-31 (ATCC-14579^T^), *Bacillus cereus* DSM-4384 (obtained from an outbreak of food poisoning) and *Bacillus cereus* 1230 strains were used as positive enterotoxigenic reference strains. *B*a*cillus subtilis* subsp. *spizizenii* DSM-347 (ATCC-6633) was used as an enterotoxin negative reference strain. All reference strains were obtained from the Leibniz Institute DSMZ-German Collection of Microorganisms and Cell Cultures, Braunschweig, Germany. The *B*. *cereus* 1230 strain was kindly provided by Professor Granum, Faculty of Veterinary Medicine and Biosciences, Norwegian University of Life Sciences (Oslo, Norway). All strains were cultivated on Columbia Sheep Blood Agar (Oxoid, Wesel, Germany) for 24 h at 37 °C.

### Bacterial growth for the RNA isolation and growth curve

A single colony of each strain was inoculated into 10 mL Brain Hearth Infusion (BHI) broth, cultured overnight at 37°C. Then, 1 mL of each culture broth was transferred to 49 mL BHI+G (BHI + 1% glucose) and incubated at 37°C. The RNA was isolated after 6 h incubation. The growth curve of *B*. *toyonensis* BCT-7112^T^ was estimated using a NanoDrop 2000c photometer (Thermo Fisher Scientific, Wesel, Germany).

### Primer and probe design

In order to detect the enterotoxin genes, six primer sets were chosen (Table 1), based on an alignment of the gene sequences publicly available for *B*. *toyonensis* BCT-7112^T^ and *B*. *cereus* DSM-31 in the National Center for Biotechnology (NCBI) GenBank (Bethesda, Massachusetts, USA). Furthermore, six 6´FAM/BHQ-1 probes were designed for the detection during qPCR (Table 1). As an endogenous control, for the relative quantitative detection of enterotoxin gene expression in the different *Bacillus* strains, the UDP-N-acetylglucosamine 2-epimerase (*udp*) gene was used. The sequence alignments of the three *Bacillus* strains (*B*. *toyonensis* BCT-7112^T^, *B*. *cereus* DSM-31, *B*. *subtilis* subsp. s*pizizenii* DSM-347) were considered for the primer design of *udp* (Table 1).

**Table 1:** Oligonucleotide primers and probes used for the quantitative reverse transcription (RT) real-time PCR.

Primer name	Gene name	Primer sequences
hblA-1-D-F	*hblA*	TGT AGT YTC ACC AGT ARC AAC
hblA-1-R		TGC AAG GTC TCT TTC ATT CTC
hblA-1-Probe		FAM-ACA ATG GAG ATA CGG CTC TTT CTG CAA-BHQ1
HblCD-F	*hblC*	CRACAGAGCAAGGTAAAACRG
HblCD-R		RGTATCAATGRCYTTCATCAGG
HblCD-Probe		FAM-TGA ATC RGC AAA AAA AGC AGC TCG TGA A-BHQ1
hblD -F	*hblD*	GGATTAACTGCAATATTAGCAGGTCA
hblD -A-R		GCTCCTCCAATAGCTGCAATAAC
hblD -Probe		FAM-ACGATTCCACAACTTCAAGCTGAAATTGAG-BBQ1
nheA-17-F2	*nheA*	GCA GTA TCT ACG AGT TGC TTC
nheA-17D-R2		GGT GAY TGT GAT CCT AAC ATT C
nheA-17-Probe		FAM- AAGGGGGGGCAAACAGAAGTGAAA–BBQ1
nheB-3D-F	*nheB*	GCA GCA GGR AAT ATT ATG CC
nheB-3D-R		GCT TTT GCT ACM GCA TGA AC
nheB-3D-Probe		FAM-AGC TGA AAG TAC AGT GAA ACA AGC TCC A-BHQ1
nheC-S-F	*nheC*	GCTGGTGGYGTACTTTGYGTAGC
nheC-A-R		GCTGTAATTGCTGCATCAATTGT
nheC-Probe		FAM-AACATGTCTTGCTGGCGGRCCRATGA-BBQ
udp1D-F	*udp*	YGC TAA TTT YAA CGT ACC TGC TTC M
udp1D-R		GTR CCR GTK CTT GTK CTD CGT GAT
udp1D-Probe		HEX-TAC CTT CTG GKC GCT CTG TTG-BHQ2

### DNA isolation

The total bacterial DNA was isolated using the DNeasy Blood & Tissue Kit (Qiagen, Darmstadt, Germany) and the protocol for Gram-positive bacteria was carried out according to the manufacturer’s instructions. The DNA concentration was estimated using a NanoDrop2000c spectrophotometer (Thermo Fisher Scientific, Wesel, Germany).

### Isolation of total RNA

The total RNA was isolated from the bacterial isolates using the RNeasy Mini Kit (Qiagen) according to the manufacturer’s instructions, including a DNAse digestion step. The RNA was eluted with 50 μL RNase-free water. The eluate was tested for possible DNA contamination and directly used in the reverse transcription reaction.

### Reverse transcription reaction

The total RNA from each sample was subjected to reverse transcription using the QuantiTect reverse transcription kit (Qiagen) according to the manufacturer’s protocol. The reverse transcription reactions were incubated at 42°C for 15 minutes, followed by a final reverse transcriptase inactivation at 95°C for three minutes.

### TaqMan-PCR conditions

The PCR was carried out in a LightCycler 96 (Roche, Penzberg, Germany) with the following setting: 1x 10 min precycle at 95°C, 40x (10 sec at 95°C, 30 sec at 60°C). The PCR premix (25 μL) contained 0.75 μL of each primer (0.3 μM final concentration), 0.25 μL probe (0.25 μM final concentration), 12.5 μL PCR 2x FastStart Essential DNA Probes Master (Roche) and 8.25 μL aqua bides. The presence of the PCR products was determined by fluorescence. For all reactions, 2.5 μL of DNA or cDNA preparation was used as a template. Each real-time reverse transcription (RT) PCR was performed in triplicate. About 5 ng of the cDNA were applied in each reaction.

### Endogenous control and relative quantitative analysis

The UDP-N-acetylglucosamine 2-epimerase (*udp*) gene was used as an endogenous control for each reaction. The RT real-time PCR data were analysed using the ΔCt method: The relative expressions of enterotoxin genes in the different strains were determined after normalization against *udp* gene (reference gene) as implemented in the Roche software application (LightCycler 96 Relative Quantification Analysis, Roche Life Science).

### Antibodies

All primary antibodies used in this study were monoclonal (mAb), produced in mice and protein A purified (mAb). They were generated and provided by Professor E. Märtlbauer, Department of Veterinary Sciences, Ludwig Maximillian University, of Munich, Germany. Antibodies were directed against the L1 (mAb 1E9) and the L2 (mAb 8B12) component of the Hbl toxin as well as the B (mAb 1E11) and C (mAb 3D6) components of the Nhe toxin. Primary antibodies directed against the B component of the Hbl toxin or the A component of the Nhe toxin were not available. The primary antibodies were detected by secondary antibodies (sab) which were horseradish-peroxidase conjugated anti-mouse IgG antibodies produced in goats (A2304, Sigma-Aldrich Biochemie GmbH, Hamburg, Germany).

### Sample preparation

Ten mL of the 6 h-cultures were centrifuged (13,000 rpm, 5 min, room temperature). Supernatants were filtered through 0.2 μm filters (Millipore) and 20 μL of 0.5 M EDTA were added to inhibit metalloproteinase (MMP) activity. Subsequently, the native supernatants were aliquoted in 2 mL portions, independently from cfu values, and stored at -20°C or used for precipitation.

As it might have been possible that concentrations of secreted enterotoxin components in the native supernatants would be too low for detection by immunoblotting, 400 μL of filtered native supernatants of each bacterial strain were mixed with 1200 μL acetone and stored at -20°C overnight for protein precipitation. After centrifugation (13,000 rpm, 5 min, room temperature), the dried protein pellets were resolved in 8 μL 2 x Laemmli buffer (4% (w/v) SDS, 20% (v/v) glycerin, 100 mmol/L Tris-HCl, pH 6.8) and 8 μL distilled water, resulting in 25-fold concentrated supernatants referring to the volume corresponding to the calculated 5x10^8^-1.5x10^9^ CFU/mL. Pure growth medium was precipitated and concentrated in parallel as the control.

### Western blotting

For the SDS-PAGE, 8 μL of the native supernatants were mixed with 8 μL 2 x Laemmli buffer. After denaturation for 5 min at 95°C, electrophoresis was performed with a sample volume of 12 μL in 12% Tris-Glycine gels (Mini Protean II, BioRad Laboratories GmbH, Germany), followed by blotting onto 0.45 μm nitrocellulose blotting membranes (Amersham Protran Premium, GE Healthcare Life Sciences, Freiburg in Breisgau, Germany). The transfer efficiency and relative loading were checked (Pierce Reversible Protein Stain Kit for nitrocellulose membranes, Thermo Fisher Scientific (Bremen) GmbH, Bremen, Germany). The optimal blocking and antibody incubation conditions are shown in Table 2. The Western blots were also incubated with secondary antibodies only for detecting non-specific signals. For the chemiluminescence detection of immunocomplexes by ChemiDoc, the membranes were treated with Supersignal West Femto Maximum Sensitivity Substrate (Thermo Scientific). All preparations and immunoblottings were performed with samples from two independent culture processes. To ensure the detection results for NheC, a third cultivation of *B*. *toyonensis* BCT-7112^T^ was conducted and prepared.

**Table 2:** Blocking and antibody incubation conditions.

Protein	Blocking 2h rt^a^	Primary antibody 4°C overnight	Secondary antibody 1 h rt
Hbl L1	3% dry milk in PBST^b^	1:100	1:20,000
		3% dry milk in PBST	3% dry milk in PBST
Hbl L2	Superblock^c^	1:100	1:20,000
		Superblock	3% dry milk in PBST
NheB	5% dry milk in PBST	1:100	1:20,000
		5% dry milk in PBST	5% dry milk in PBST
NheC	5% dry milk in PBST	1:200(pellet, conc. supernatant)	1:20,000
		1:100 (pure supernatant)	5% dry milk in PBST
		5% dry milk in PBST	

^a^ Room temperature

^b^ Phosphate buffered saline containing 0.1% Tween 20 (Thermo Scientific)

^c^ Blocking buffer in PBS containing 0.1% Tween 20

The native or concentrated pure growth medium showed no detectable membrane staining at all, enabling the employment of sample membrane staining after blotting as the relative loading control. This approach was used because the protein quantification of the supernatants using the conventional Bradford method (BioRad) was not possible due to the very low protein levels and high background probably caused by the intrinsic culture medium components in all tested strains.

### Purification and concentration of supernatants for sequencing

Due to a need for higher purity of concentrated supernatants for sequencing by LC-MS (liquid chromatography–mass spectrometry), Amicon filters were used for the concentration (performed by Dr. J. Meens, Institute of Microbiology, University of Veterinary Medicine Hannover, Foundation, Germany). Six mL of the supernatant were centrifuged (5,000 *g*, 4°C, 20 min) with a 3 kDa Amicon Ultra-15 filter resulting in a residual volume of ~1 mL concentrated supernatant. This residual volume was diluted with 8 mL phosphate buffered saline (PBS), centrifuged (5,000 *g*, 4°C, 30 min) and the same step was repeated again. Subsequently, the resulting concentrate (~2 mL) was centrifuged (14,000 *g*, 4°C, 10 min) with a 10 kDa Amicon Ultra-0.5 filter, yielding a final volume of 30–40 μL for each concentrated supernatant (= 150-200-fold concentration). These concentration levels corresponded to the calculated 9x10^9^-1.2x10^10^ CFU/mL, 3-4x10^9^ CFU/mL and 4.5-6x10^9^ CFU/mL for *B*. *cereus* 1230, *B*. *subtilis* subsp. *spizizenii* DSM-347 and *B*. *toyonensis* BCT-7112^T^, respectively.

### Sequencing and analysis

Three μL of the Amicon filter concentrated supernatants were filled up with 8.5 μL 2 x Laemmli buffer and distilled water, denatured for 5 min at 95°C and subsequently alkylated by adding acrylamide up to 2% (v/v) for 30 min at room temperature. After electrophoresis of a 12 μL sample per lane, the 12% Tris-Glycine gel was stained over night with a Coomassie staining solution, discoloured to visualise protein bands and transported in distilled water together with a respective Western blot image for orientation to the sequencing laboratory (Professor A. Pich, Institute of Toxicology, Research Core Unit–Proteomics, Hannover Medical School, Hannover, Germany). The gel areas reflecting the putative NheC positive bands were cut and a further sample processing was performed as described [11]. Dried peptide extracts were redissolved in 30 μL 2% acetonitrile (ACN), 0.1% TFA with shaking at 800 rpm for 20 min. After centrifugation at 20,000 *g*, aliquots of 12.5 μL each were stored at -20°C.

### LC-MS analysis

The peptide samples were separated with an ultra-high pressure nano-flow rapid separation liquid chromatography system (RSLC, Thermo Scientific) equipped with a trapping column (3 μm C18 particle, 2 cm in length, 75 μm ID, Acclaim PepMap, Thermo Scientific) and a 50 cm long separation column (2 μm C18 particle, 75 μm ID, Acclaim PepMap, Thermo Scientific). The peptide mixtures were injected, enriched and desalted on the trapping column at a flow rate of 6 μL/min with 0.1% TFA for 5 min. The trapping column was switched online with the separating column and peptides were eluted with a multi-step binary gradient: Linear gradient of buffer B (80% ACN, 0.1% formic acid) in buffer A (0.1% formic acid) from 4% to 25% in 30 min, 25% to 50% in 10 min, 50% to 90% in 5 min and 10 min at 90% buffer B, respectively. The column was reconditioned to 4% buffer B in 15 min. The flow rate was 250 nL/min and the column temperature was set at 45°C. The RSLC system was coupled online via a Nano Spray Flex Ion Source II (Thermo Scientific) to a Linear Trap Quadropole (LTQ) Orbitrap Velos mass spectrometer. Metal-coated fused-silica emitters (SilicaTip, 10 μm i.d., New Objectives Inc., Woburn, Massachusetts, USA) and a voltage of 1.3 kV were used for the electrospray. Overview scans were acquired at a resolution of 60k in a mass range of m/z 300–1600 in the orbitrap analyzer and stored in profile mode. The top ten most intensive ions with charges of two or three and a minimum intensity of 2,000 counts were selected for collision-induced dissociation (CID) fragmentation with a normalised collision energy of 38.0, an activation time of 10 ms and an activation Q of 0.250 in the LTQ. Fragment ion mass spectra were recorded in the LTQ at normal scan rate and the centroid m/z value and intensity pairs were stored. Active exclusion was activated so that ions fragmented once were excluded from further fragmentation for 70 s within a mass window of 10 ppm of the specific m/z value.

Raw data were processed using Proteome Discoverer (Thermo Scientific) and a home *Bacillus cereus* database containing common contaminants. Proteins were stated, identified by a false discovery rate of 0.01 at protein and peptide level. For further proof of the occurrence of NheC, the identified proteins were analysed using SwissProt/UniProt and NCBI databases by alignments. Subsequently, sequences were aligned with the putative epitope “TNMTETIDAA” of antibody mAb 3D6 [12].

## Results

### Detection of the enterotoxin genes using PCR

All toxin gene sequences could be confirmed at DNA level for the *B*. *toyonensis* BCT-7112^T^ and the *B*. *cereus* strains. *Bacillus subtilis* subsp. *spizizenii* DSM-347 showed negative results for the toxin genes. The DNA sequence for *udp* gene was detected in all investigated strains with a Ct value between 19 and 23 (S1 Table).

### RT real-time PCR results

The normalised expression levels of the toxin genes *hblA*, *hblC*, *hblD* and *nheA*, *nheB*, *nheC* expressed as ΔCt mean ± SEM are shown in Table 3 (raw data are shown in S2 and S3 Tables). The results showed that the expression levels of the Hbl and Nhe toxin genes were considerably higher in all *B*. *cereus* reference strains compared to *B*. *toyonensis* BCT-7112^T^. The expression of *hblC* was absent in *B*. *toyonensis* BCT-7112^T^.

**Table 3:** The quantification analysis data (ΔCt) of the Hbl and Nhe toxin genes expression after normalisation with the *udp* reference gene. The toxin gene expression of *hblC* was absent in *B*. *toyonensis* BCT-7112^T^ (n = 3).

*Bacillus* Strain Name	ΔCt mean (±SEM^a^)					
	*hblA*	*hblC*	*hblD*	*nheA*	*nheB*	*nheC*
***B*. *toyonensis* BCT-7112**^**T**^	**0.31** (0.18)	-	**0.13** (0.02)	**0.07** (0.00)	**2.03** (0.74)	**0.11** (0.02)
***B*. *cereus* 1230**	**1.35** (0.80)	**2.64** (0.58)	**0.28** (0.12)	**0.43** (0.04)	**46.48** (9.73)	**0.32** (0.19)
***B*. *cereus* DSM-4384**	**1.41** (1.06)	**1.94** (0.70)	**0.46** (0.26)	**2.26** (0.56)	**74.49** (34.26)	**3.07** (2.61)
***B*. *cereus* DSM-31**	**0.44** (0.17)	**4.09** (3.25)	**0.21** (0.08)	**0.63** (0.34)	**66.13** (55.81)	**0.62** (0.16)
***B*. *subtilis* subsp. *spizizenii* DSM-347**	-	-	-	-	-	-

^a^Standard error of the mean

### Detection of secreted enterotoxin compounds

**Fig 1:**
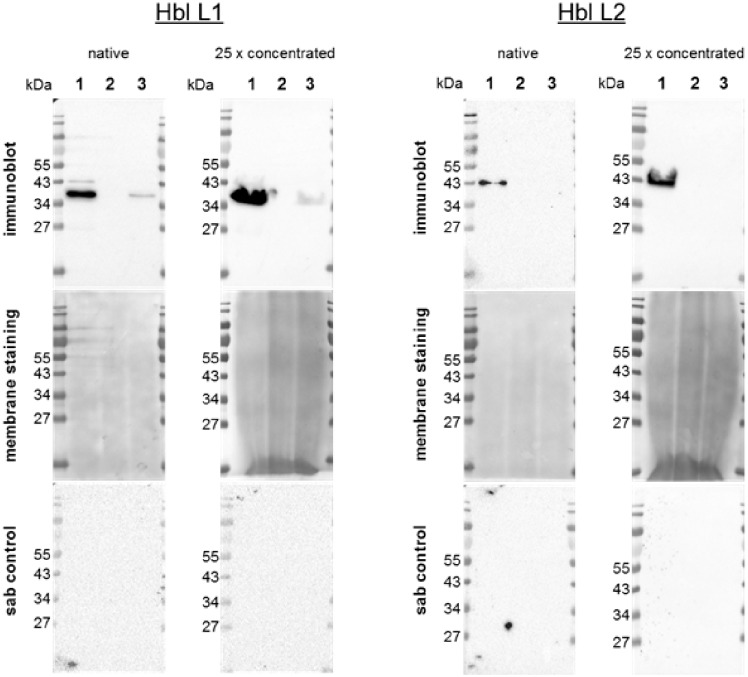
Representative immunoblottings, membrane staining and sab controls of Hbl L1 and Hbl L2 detection. Native and concentrated (25-fold) supernatants prepared from (1) *B*. *cereus* 1230 as positive control, (2) *B*. *subtilis* subsp. *spizizenii* DSM-347 as negative control and (3) *B*. *toyonensis* BCT-7112^T^. Non-specific protein staining of membranes as loading control is shown as well as respective sab controls. Relevant molecular weights are indicated.

In both native and 25-fold concentrated supernatants from *B*. *cereus* 1230 and *B*. *toyonensis* BCT-7112^T^, Hbl L1 was detected. The respective band detected in supernatants produced by *B*. *toyonensis* BCT-7112^T^ was weaker than that observed in *B*. *cereus* 1230 supernatants (Fig 1). No signal was observable in the supernatants from *B*. *subtilis* subsp. *spizizenii* DSM-347. Additional bands in *B*. *cereus* 1230 supernatants were present at ~43 and ~26 kDa positions. The sab control showed no non-specific binding. Compound Hbl L2 was only detected in the native and concentrated supernatants from *B*. *cereus* 1230 (Fig 1). No signals were observable in the native or 25-fold concentrated supernatants from *B*. *toyonensis* BCT-7112^T^ or *B*. *subtilis* subsp. *spizizenii* DSM-347. The secondary antibody controls with the native supernatants of *B*. *cereus* 1230 and *B*. *toyonensis* BCT-7112^T^ as well as with the concentrated supernatant of *B*. *cereus* 1230 showed no non-specific signals.

**Fig 2:**
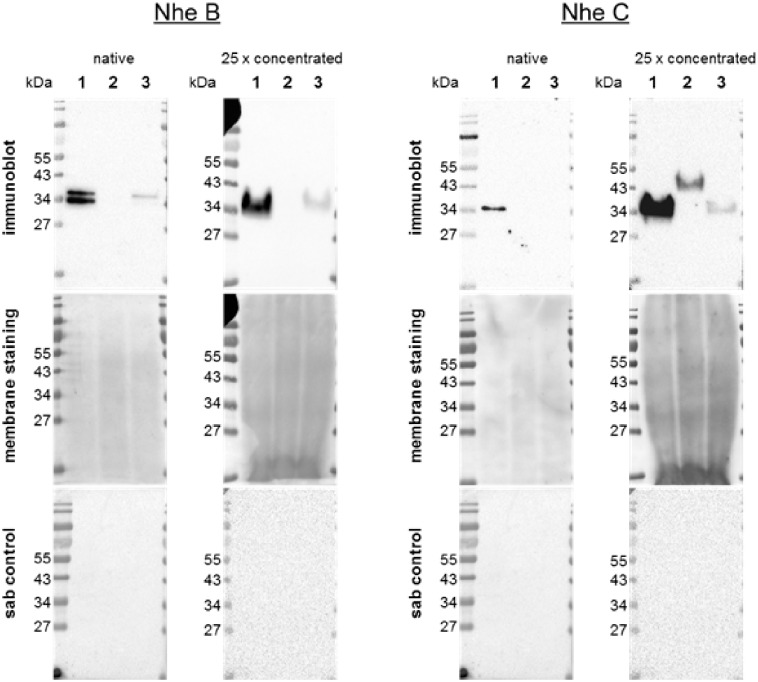
Representative immunoblottings, membrane staining and sab controls of NheB and NheC detection. Native and concentrated (25-fold) supernatants prepared from (1) *B*. *cereus* 1230 as positive control, (2) *B*. *subtilis* subsp. *spizizenii* DSM-347 as negative control and (3) *B*. *toyonensis* BCT-7112^T^. Non-specific protein staining of membranes as loading control as well as respective sab controls are shown. The sab controls of both compounds are identical because the blocking and incubation conditions were performed identically. Relevant molecular weights are indicated.

All samples were positive for the compound NheB in *B*. *cereus* 1230 and *B*. *toyonensis* BCT-7112^T^. The supernatants showed a band at the ~40 kDa position in both strains and an additional band lower than 34 kDa in *B*. *cereus* 1230 (Fig 2). However, the band detected at the ~40 kDa position in the supernatants from *B*. *toyonensis* BCT-7112^T^ was weaker than that observed in the supernatants from *B*. *cereus* 1230. The samples of *B*. *subtilis* subsp. *spizizenii* DSM-347 were negative as were the sab controls of all strains. In native supernatants, only *B*. *cereus* 1230 showed a positive signal for NheC (Fig 2). A respective band for NheC was not detected in the native supernatant from *B*. *toyonensis* BCT-7112^T^. A weak positive signal for NheC was observed in the 25-fold concentrated supernatants of *B*. *toyonensis* BCT-7112^T^. However, a positive signal in *B*. *subtilis* subsp. *spizizenii* DSM-347 at the ~45 kDa position was also observed at the 25-fold concentration of supernatants.

### Sequencing of NheC positive bands

**Fig 3:**
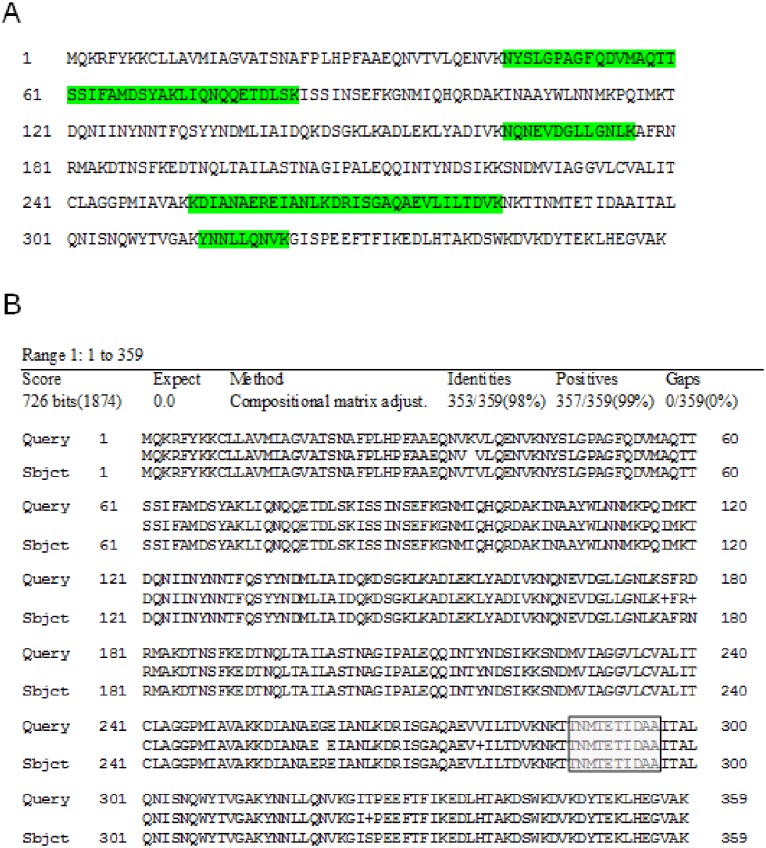
Sequencing of *B*. *cereus* NheC positive band. (A) NCBI Ref. Seq. NP_831584.1 (enterotoxin C) from *B*. *cereus* ATCC-14579^T^ database. Appropriate peptides found by sequencing are highlighted in green. (B) NCBI GenBank No. CAB53340.2 (enterotoxin C–*B*. *cereus* NVH 1230–88) as query and Acc. No. NP_831584.1 (enterotoxin C–*B*. *cereus* ATCC-14579^T^) as subject. The putative epitope of the used antibody is highlighted in grey.

To exclude false positive signals for NheC, samples of the supernatants of all strains were prepared and concentrated for sequencing using the LC-MS method. Analyses of the results showed seven unique sequences of detected peptides in the NheC antibody positive gel area of *B*. *cereus* 1230. They shared 100% similarity with individual peptide sequences, present in proteins, identified as belonging to different bacterial strains ranging from 31 to 250 strains for each peptide. Most of them belonged to the *Bacillus cereus* group. Among the identified proteins, the UniProtKB Accession No. Q813Q6 (non-expressed enterotoxin C) shared 100% similarity with all detected peptide sequences (Fig 3A). The respective protein was identified as belonging to the *Bacillus cereus* type strain (ATCC 14579^T^ / DSM 31 / JCM 2152 / NBRC 15305 /NCIMB 9373 / NRRL B-3711) and corresponded to the NCBI Reference Sequence: NP_831584.1 (enterotoxin C). The detected peptides also showed high similarities to respective peptide sequences in a protein identified with UniProtKB Accession No. Q9S3N2 (enterotoxin C) as belonging to *Bacillus cereus* NVH 1230–88, corresponding to the NCBI GenBank No. CAB53340.2 (enterotoxin C). The NCBI database alignment of both identified enterotoxin NheC proteins (*B*. *cereus* NHV 1230–88 and *B*. *cereus* ATCC 14579) showed 98% similarity (Fig 3B).

**Fig 4:**
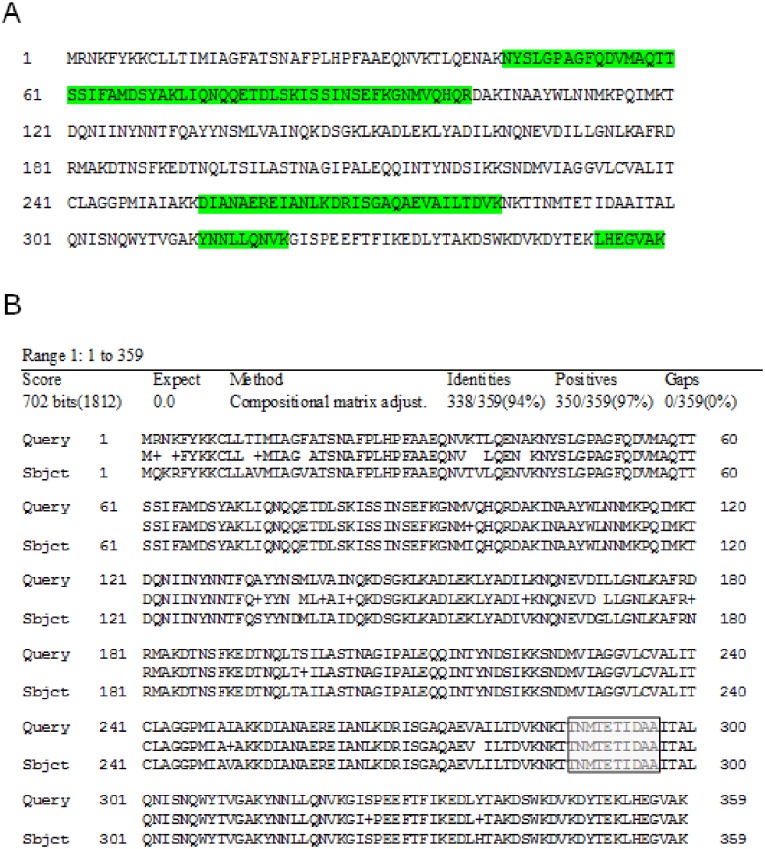
Sequencing of *B*. *toyonensis* BCT-7112^T^ NheC positive band. (A) NCBI GenBank No. AHA10308.1 (enterotoxin C) from *B*. *toyonensis* BCT-7112^T^ database. Appropriate peptides found by sequencing are highlighted in green. (B) NCBI GenBank No. AHA10308.1 (enterotoxin C–*B*. *toyonensis* BCT-7112^T^) as query and Acc. No. NP_831584.1 (enterotoxin C–*B*. *cereus* ATCC-14579^T^) as subject. The putative epitope of the NheC antibody is highlighted in grey.

Nine unique sequences of detected peptides in the NheC antibody positive gel area of the *B*. *toyonensis* BCT-7112^T^ lane shared 100% similarity to respective peptide sequences present in the protein identified with UniProtKB Accession No. U5ZZA5 (enterotoxin C) as belonging to *Bacillus toyonensis* BCT-7112^T^ that corresponds with NCBI GenBank No. AHA10308.1 (Fig 4A).

The NCBI database alignment of the identified protein, belonging to *Bacillus toyonensis* BCT-7112^T^, showed 94% similarity to the *B*. *cereus* ATCC 1479 enterotoxin C sequence (NCBI Ref. Seq. NP_831584.1) (Fig 4A). The putative epitope of the used NheC antibody TNMTETIDAA was also present in the identified protein at the same position (amino acids 287–296).

To identify the band caused by the NheC antibody in the supernatant of the negative control, all proteins identified in the *B*. *subtilis* subsp. *spizizenii* DSM-347 lane in the area around 45 kDa (*B*. *subtilis* subsp. *spizizenii* database) were aligned with the epitope sequence TNMTETIDAA using the SIM alignment tool (www.web.expasy.org/sim/). The alignments were also performed with the identified proteins in the *B*. *subtilis* subsp. *spizizenii* DSM-347 lane using the *B*. *cereus* database. However, the complete epitope was found in none of the identified proteins. The discovered similarities accounted at the most for 60% of the epitope sequence and were probably too low to explain the positive signals in the Western blotting by non-specific binding of the used antibody. Therefore, we were not able to identify the source of the NheC antibody binding to the concentrated supernatants of *B*. *subtilis* subsp. *spizizenii* DSM-347.

## Discussion

The long history of safe use of *B*. *toyonensis* BCT-7112^T^ as a feed additive in animal nutrition has recently been questioned. DNA investigations [7, 13, 14] revealed that the strain *B*. *toyonensis* BCT-7112^T^ indeed carries the genes coding for two enterotoxins produced by *B*. *cereus* strains, namely haemolysin BL (Hbl) and non-haemolytic enterotoxin (Nhe) [15, 16]. Since the results of DNA investigations alone do not permit conclusions on the functionality or non-functionality of the expressed proteins, the hypothesis may still be that the genes coding for the Hbl and Nhe enterotoxins might not be functional in *B*. *toyonensis* BCT-7112^T^ [14]. In this context, the European Food Safety Authority (EFSA) as well questioned the quality and reliability of a Western blot assay and stated that the non-functionality of the genes coding for Hbl and Nhe enterotoxins was not demonstrated yet [17]. In order to clarify this and in order to avoid the risk of false negative PCR results, new real-time PCR assays for detecting the main enterotoxin genes *hbl* and *nhe* were developed and evaluated, based on the sequences of *B*. *toyonensis* BCT-7112^T^ and *B*. *cereus* DSM-31. At DNA level, the developed real-time PCR showed positive signals with all six gene components of Hbl and Nhe toxins in all tested strains. However, expression levels of the genes *hblA*, *hblD*, *nheA*, *nheB* and *nheC* detected in *B*. *toyonensis* BCT-7112^T^ were lower or, in the case of *hblC*, absent in *B*. *toyonensis* BCT-7112^T^ compared to the *B*. *cereus* positive reference strains. This finding suggests that although the enterotoxin genes are present in the genome of *B*. *toyonensis* BCT-7112^T^, they do not seem to be equally active.

To verify this assumption, further investigations were conducted at protein level, focussing on the ability to produce the enterotoxin components. The use of membrane staining enabled the estimation of the sample loadings as a prerequisite for evaluating expression or secretion of the enterotoxin components in *B*. *toyonensis* BCT-7112^T^ compared with the positive control *B*. *cereus* 1230 reference strain. Despite the very low total protein concentrations in native supernatants and the high concentration of medium components impeding protein quantification of samples by the Bradford method, the presence of the proteins in the Western blot analyses could be shown. The acetone-mediated concentration of the supernatants resulted in an overloading of total protein per lane, presumably caused by parallel concentration of medium ingredients which was observable on the stained membranes. This negatively affected the forming of clear and distinct bands compared with the native supernatants. Nevertheless, the detection of enterotoxin components in the positive control was more pronounced, this result strengthening the reliability of negative results allowing the detection of very low secretory levels. The specificity of the positive enterotoxin component detections in the supernatants could be demonstrated by the sab controls which were completely negative. Due to the lack of specific antibodies directed against the Hbl B component, its positive expression could not be verified in our study. Nevertheless, the absence of the expression of the gene *hblC* and the Hbl L2 component of Hbl enterotoxin indicates that *B*. *toyonensis* BCT-7112^T^ does not have the ability to produce a biological active haemolysin BL enterotoxin. The expression and secretion of NheB by *B*. *toyonensis* BCT-7112^T^ was also confirmed in this study. Similar to Hbl B, the expression of NheA could not be examined due to the lack of a specific antibody in this present study. For both Hbl and Nhe enterotoxin complexes, the combination of their respective components is necessary in order to develop their enterotoxic potential [5, 6].

The detection of NheC in the native supernatant produced by ~3x10^7^ CFU/mL *B*. *toyonensis* BCT-7112^T^ in 6 h cultures was negative. It has been described that the Nhe toxin requires the expression of its individual enterotoxin components Nhe A, B and C in a certain ration (10:10:1) and a specific binding order for full in vitro cytotoxicity [4]. First, the components NheB and NheC need to be combined, binding to the cell surface, and a conformational change occurs that allows subsequent binding of NheA, resulting in cell lysis [18]. However, a weak band representing NheC could be detected in the 25-fold concentrated supernatants taken from *B*. *toyonensis* BCT-7112^T^, corresponding to calculated 7.5x10^8^ CFU/mL. The analysed protein shared a 94% similarity with the non-haemolytic enterotoxin component C described for the *B*. *cereus* 1230 positive control. Nevertheless, whether or not this secreted protein in *B*. *toyonensis* BCT-7112^T^ 25-fold concentrated supernatants possesses the same functional properties as that of the *B*. *cereus* 1230 cannot be estimated on the basis of our results. However, in order to completely exclude the toxigenic potential under *in vivo* conditions respective experiments have to be carried out.

The source of the NheC positive signals at ~45 kDa molecular weight in the supernatants of the NheC negative B. *subtilis* strain could not be identified. However, it might be possible that the NheC antibody recognised a protein with a concentration too low for detection by the used sequencing procedure. Thus, it could not be found in the database search. Alternatively, it is possible that the detected signal represents a protein that has not been described or included in the *B*. *subtilis* database yet.

Altogether, the non-toxigenic and non-pathogenic character of *B*. *toyonensis* BCT-7112^T^ is supported by a number of *in vivo* safety studies such as enterotoxicity studies (e.g., ileal loop accumulation tests in rabbits), acute toxicity studies, subchronic toxicity studies, chronic toxicity studies, genotoxicity studies, irritation studies and a clinical evaluation in humans [19], and *in vitro* safety studies such as cytotoxicity studies in neonatal pig small intestinal cells, mature pig small intestinal cells, human foetal small intestinal cells [20] and Vero cells [21].

To conclude, the results of the present study showed that the enterotoxin expression and protein levels of *B*. *toyonensis* BCT-7112^T^ were clearly lower or absent compared to the *B*. *cereus* reference strains. Although the ability to form enterotoxins functionally can be considered to be lower compared to the *B*. *cereus* reference strain, it cannot be completely excluded under *in vivo* conditions. This fact has to be taken into account regarding the application safety of these probiotic microorganisms. Thus, *in vivo* experiments have to be carried out for a final evaluation.

## Supporting information

S1 Table Detected Ct values for *nheA*, *nheB* and *hblA* enterotoxin genes and *udp* DNA in the *Bacillus toyonensis* BCT 7112^T^ strain and the three toxin-positive strains as well as *Bacillus subtilis* subsp. *spizizenii* DSM-347 as the toxin negative strain. (DOCX)

S2 Table Supplementary data for Table 3. The quantification analysis data (ΔCt) of the Hbl toxin gene expression after normalization with the *udp* reference gene. The toxin gene expression of *hblC* was absent in *B*. *toyonensis* BCT-7112^T^ (n = 3).
(DOCX)

S3 Table Supplementary data for Table 3. The quantification analysis data (ΔCt) of the Nhe toxin gene expression after normalization with the *udp* reference gene (n = 3).
(DOCX)
