# Supplementary material for: Evaluation of enterotoxin gene expression and enterotoxin production capacity of the probiotic strain Bacillus toyonensis BCT-7112T
Source: PLoS One. 2019 Apr 25;14(4):e0214536. doi: 10.1371/journal.pone.0214536 (PMC6483178; doi:10.1371/journal.pone.0214536)
Supplement: S1 Table — (DOCX) [file pone.0214536.s001.docx]

**S1** **Table. Detected Ct values for *nheA*, *nheB* and *hblA* enterotoxin genes and *udp* DNA in the *Bacillus toyonensis* BCT 7112^T^ strain and the three toxin-positive strains as well as *Bacillus* *subtilis* subsp*. spizizenii* DSM-347 as the toxin negative strain.**

| ***Bacillus* Strain Name** | **Ct values** | | | | | | |
| --- | --- | --- | --- | --- | --- | --- | --- |
|  | ***hblA*** | ***hblC*** | ***hblD*** | ***nheA*** | ***nheB*** | ***nheC*** | ***udp*** |
| ***B. toyonensis* BCT-7112^T^** | 19.17 | 18.91 | 16.98 | 12.25 | 14.08 | 16.11 | 18.89 |
| ***B. cereus* 1230** | 24.19 | 20.13 | 18.41 | 16.01 | 13.23 | 16.71 | 20.66 |
| ***B. cereus* DSM-4384** | 24.01 | 19.84 | 17.96 | 11.54 | 12.85 | 19.05 | 20.61 |
| ***B. cereus* DSM-31** | 23.61 | 18.72 | 16.94 | 14.88 | 12.45 | 16.76 | 23.18 |
| ***B. subtilis* subsp. *spizizenii* DSM-347** | - | - | - | - | - | - | 22.37 |
