# Supplementary material for: Evaluation of enterotoxin gene expression and enterotoxin production capacity of the probiotic strain Bacillus toyonensis BCT-7112T
Source: PLoS One. 2019 Apr 25;14(4):e0214536. doi: 10.1371/journal.pone.0214536 (PMC6483178; doi:10.1371/journal.pone.0214536)
Supplement: S2 Table — The quantification analysis data (ΔCt) of the Hbl toxin gene expression after normalization with the udp reference gene. The toxin gene expression of hblC was absent in B. toyonensis BCT-7112T (n = 3). (DOCX) [file pone.0214536.s002.docx]

**S2 Table. Supplementary data for Table 3. The quantification analysis data (ΔCt) of the Hbl toxin gene expression after normalization with the *udp* reference gene. The toxin gene expression of *hblC* was absent in *B. toyonensis* BCT-7112^T^ (n=3).**

| ***Bacillus* Strain Name** | **ΔCt** | | | | | | | | |
| --- | --- | --- | --- | --- | --- | --- | --- | --- | --- |
|  | ***hblA*** | | | ***hblC*** | | | ***hblD*** | | |
| **n** | 1 | 2 | 3 | 1 | 2 | 3 | 1 | 2 | 3 |
| ***B. toyonensis* BCT-7112^T^** | 0.240 | 0.655 | 0.044 | - | - | - | 0.090 | 0.170 | 0.121 |
| ***B. cereus* 1230** | 0.953 | 2.888 | 0.202 | 3.784 | 2.266 | 1.866 | 0.518 | 0.144 | 0.193 |
| ***B. cereus* DSM-4384** | 0.607 | 3.506 | 0.121 | 3.010 | 0.611 | 2.204 | 0.986 | 0.155 | 0.250 |
| ***B. cereus* DSM-31** | 0.497 | 0.697 | 0.130 | 10.556 | 0.270 | 1.434 | 0.363 | 0.141 | 0.135 |
