# Supplementary material for: Evaluation of enterotoxin gene expression and enterotoxin production capacity of the probiotic strain Bacillus toyonensis BCT-7112T
Source: PLoS One. 2019 Apr 25;14(4):e0214536. doi: 10.1371/journal.pone.0214536 (PMC6483178; doi:10.1371/journal.pone.0214536)
Supplement: S3 Table — The quantification analysis data (ΔCt) of the Nhe toxin gene expression after normalization with the udp reference gene (n = 3). (DOCX) [file pone.0214536.s003.docx]

**S3 Table. Supplementary data for Table 3. The quantification analysis data (ΔCt) of the Nhe toxin gene expression after normalization with the *udp* reference gene (n=3).**

| ***Bacillus* Strain Name** | **ΔCt** | | | | | | | | |
| --- | --- | --- | --- | --- | --- | --- | --- | --- | --- |
|  | ***nheA*** | | | ***nheB*** | | | ***nheC*** | | |
| **n** | 1 | 2 | 3 | 1 | 2 | 3 | 1 | 2 | 3 |
| ***B. toyonensis* BCT-7112^T^** | 0.070 | 0.076 | 0.071 | 0.901 | 3.434 | 1.765 | 0.076 | 0.110 | 0.149 |
| ***B. cereus* 1230** | 0.356 | 0.493 | 0.448 | 65.799 | 34.776 | 38.854 | 0.250 | 0.688 | 0.037 |
| ***B. cereus* DSM-4384** | 2.346 | 3.182 | 1.248 | 55.330 | 27.096 | 141.044 | 0.268 | 0.646 | 8.282 |
| ***B. cereus* DSM-31** | 0.264 | 0.314 | 1.301 | 1.803 | 177.294 | 19.293 | 0.314 | 0.712 | 0.829 |
